# Variation in chronic radiation exposure does not drive life history divergence among *Daphnia *populations across the Chernobyl Exclusion Zone

**DOI:** 10.1002/ece3.4931

**Published:** 2019-02-03

**Authors:** Jessica Goodman, David Copplestone, Gennady V. Laptev, Sergey Gashchak, Stuart K. J. R. Auld

**Affiliations:** ^1^ Biological and Environmental Sciences, Faculty of Natural Sciences Stirling University Stirling UK; ^2^ Ukrainian HydroMeteorological Institute Kiev Ukraine; ^3^ International Chornobyl Center 11 Slavutych Kyiv Region Ukraine

**Keywords:** *Daphnia pulex*, life history, mutation, radiation

## Abstract

Ionizing radiation is a mutagen with known negative impacts on individual fitness. However, much less is known about how these individual fitness effects translate into population‐level variation in natural environments that have experienced varying levels of radiation exposure. In this study, we sampled genotypes of the freshwater crustacean, *Daphnia pulex*, from the eight inhabited lakes across the Chernobyl Exclusion Zone (CEZ). Each lake has experienced very different levels of chronic radiation exposure since a nuclear power reactor exploded there over thirty years ago. The sampled *Daphnia* genotypes represent genetic snapshots of current populations and allowed us to examine fitness‐related traits under controlled laboratory conditions at UK background dose rates. We found that whilst there was variation in survival and schedules of reproduction among populations, there was no compelling evidence that this was driven by variation in exposure to radiation. Previous studies have shown that controlled exposure to radiation at dose rates included in the range measured in the current study reduce survival, or fecundity, or both. One limitation of this study is the lack of available sites at high dose rates, and future work could test life history variation in various organisms at other high radiation areas. Our results are nevertheless consistent with the idea that other ecological factors, *for example *competition, predation or parasitism, are likely to play a much bigger role in driving variation among populations than exposure to the high radiation dose rates found in the CEZ. These findings clearly demonstrate that it is important to examine the potential negative effects of radiation across wild populations that are subject to many and varied selection pressures as a result of complex ecological interactions.

## 1 INTRODUCTION

Populations are constantly challenged with selection from competitors, predators, and parasites (Auld & Brand, 2017; Ball & Baker, 1996; McLaughlin, Hellmann, Boggs, & Ehrlich, 2002). An increase in human activities means that natural populations are also at a higher risk of sudden, dramatic changes to their environment (from events such as oil spills, chemical releases, and climate change) (Bickham, Sandhu, Hebert, Chikhi, & Athwal, 2000; Husseneder, Donaldson, & Foil, 2016; McLaughlin et al., 2002; Riffaut, McCoy, Tirard, Friesen, & Boulinier, 2005), which can have detrimental impacts on individuals and thus populations (*e.g., *Bickham & Smolen, 1994; Santos et al., 2013).

Nuclear accidents such as those at Chernobyl and Fukushima are prime examples of human‐induced dramatic environmental change. These accidents have resulted in widespread radioactive contamination of the surrounding areas. The levels of ionizing radiation across these areas show considerable variation both over space, due to heterogeneity in radionuclide deposition and over time, as a result of radionuclide decay (Saxen et al., 1987; Saito et al., 2015). Whilst negative effects of radiation on individuals are known (Breimer, 1988; Morgan, 2003a, 2003b; von Sonntag,^2007^), it is difficult to extrapolate effects on individuals to the level of the population (Bréchignac,^2017^). These difficulties arise because of two key issues: first, organisms living within high radiation environments (> 420 μGy/h) (Hinton et al., 2007) could exhibit a lower overall mean fitness due to physiological stress (Kimura & Maruyama, 1966). Second, strong selection for radiation‐tolerant individuals could reduce differences in mean fitness between high‐ and low‐radiation populations (Esnault, Legue, & Chenal, 2010; Galván et al., 2014) and thus mask the negative effects of radiation on individuals. Indeed, strong selection for radiation‐tolerant phenotypes may explain how some natural populations can persist in high radiation environments (Baker et al., 1996; Murphy, Nagorskaya, & Smith, 2011).

Ionizing radiation also generates mutations, which are the founding source of all genetic variation (Haldane, 1937; Kimura & Maruyama, 1966). Variation in fitness‐related traits in contemporary populations may therefore be exacerbated by exposure to radiation in the Chernobyl Exclusion Zone (CEZ). However, ionizing radiation can also exert selection on populations, and the evolution of radiation tolerance may drive depletion in population genetic variation. Both the mean and variance in fitness‐related traits can give us valuable insight into the balance between mutation (which causes increased variance and lower mean fitness: Kimura & Maruyama, 1966) and selection (reduced variance with either no difference or increased mean fitness: Haldane, 1937; Crow,^1970^). It is, however, important to note that whilst mutation is the ultimate source of all genetic variation, radiation is just one of many possible agents of selection. Ecological factors such as parasitism, predation, and competition are known to have impacts on population fitness and may outweigh any effects of radiation in wild populations (Auld et al., 2013; Brockelman, 1975; Creel & Christianson, 2008; Lehmann, 1993). Moreover, these ecological factors can influence fitness indirectly *for example, *by selecting on the predators, parasites, or prey of the focal organism rather than on the focal organism itself (Ball & Baker, 1996; Reznick, Bryga, & Endler, 1990). Still, by quantifying trait variation among organisms collected across a gradient of chronic radiation dose, we can nevertheless test whether radiation exposure plays the dominant role in shaping fitness at the population level.

The CEZ provides a useful natural laboratory to test how variation in ionizing radiation shapes life histories and fitness across wild populations. The Chernobyl accident caused an estimated release of approximately 1.85 × 10^18^ Bq of radioactive material (IAEA, 2006). Initially, the radiation doses were dominated by short‐lived and highly damaging radionuclides such as ^133^Xe, ^131^I, and ^140^Ba (NEA, 2002) distributed heterogeneously across Chernobyl, with profound negative consequences for surrounding wildlife (UNSCEAR, 2008). After the rapid decay of these short‐lived radionuclides, longer‐lived radionuclides such as ^137^Cs and ^90^Sr remained, becoming more dominant (Kryshev, 1995; Nazarov & Gudkov, 2008). The spatial heterogeneity in chronic radiation across the CEZ (Figure 2, Supporting Information Table S1) provides an opportunity to test for dose‐dependent effects of ionizing radiation on natural populations.

There are, however, major challenges associated with testing the fitness impacts of radiation exposure using natural populations. For example, individuals frequently move across a patchy landscape of radiation, making it difficult to estimate the overall absorbed dose they experience (Hinton et al., 2007). We overcame this problem by studying *Daphnia pulex*, a freshwater crustacean that inhabits discrete ponds and lakes with low interpopulation migration (Haag, Riek, Hottinger, Pajunen, & Ebert, 2006) where we could obtain reliable estimates of absorbed radiation dose. *Daphnia pulex* provides other advantages: it reproduces both sexually and asexually, where most reproduction is asexual, but sex is required to produce hardy resting eggs that can survive the winter (Zaffagnini, 1987). By collecting *Daphnia *from lakes and ponds across the Chernobyl area, we were able to obtain a genetic snapshot of populations that have experienced very different levels of chronic radiation (from <0.1 to over 180 µGy/h) and conduct a common garden experiment where fitness‐related traits could be quantified under UK natural background radiation levels. Specifically, we measured survival and asexual reproduction over the course of the *Daphnia *lifespan. We then used these data to calculate the instantaneous rate of population increase, *r,* for each genotype (a useful proxy for overall fitness).

In this study, we explore how *Daphnia* life history traits reflect evolutionary responses to long‐term radiation exposures across the CEZ, with particular focus on the opposing processes of selection versus mutational input. We tested whether selection played a primary role in shaping populations by examining whether the variation associated with population fitness (instantaneous growth rate, *r*) declines with dose rate. We also examined whether radiation reduced mean population fitness by testing whether *Daphnia* fitness declines with dose rate, as would be consistent with previous studies that have demonstrated laboratory exposure to radiation reduces invertebrate fitness (Nohara et al., 2014; Parisot, Bourdineaud, Plaire, Adam‐Guillermin, & Alonzo, 2015; Sarapultseva & Gorski, 2013).

## 2 MATERIALS AND METHODS

### 2.1 Study system

*Daphnia *are sensitive to environmental change and have thus proven an excellent model for ecotoxicology (Flaherty & Dodson, 2005; Pace et al., 2004); indeed, *Daphnia* reproduction is used as an OECD test species for testing the toxicity of various chemicals and pollutants (OECD, 2012). Furthermore, immigration of *Daphnia* between populations is rare and is generally limited to the diapausing stage of their reproductive lifecycle (Haag et al., 2006), so individual *Daphnia *phenotypes are likely to have been shaped primarily by the immediate environment. Finally, *Daphnia* are cyclical parthenogens, whereby they reproduce asexually throughout the spring/summer and sexually to produce resting eggs which remain dormant over the Autumn/Winter (Alekseev & Lampert, 2001; Decaestecker, Meester, & Mergeay, 2009). This mixed reproductive mode means one can take advantage of their asexual reproductive stage to take genetic snapshots of wild populations and then examine clonal lines in replicated common garden experiments under controlled conditions (*e.g., *Auld et al., 2013).

### 2.2 Field collections and radiation dosimetry

**Figure 1:**
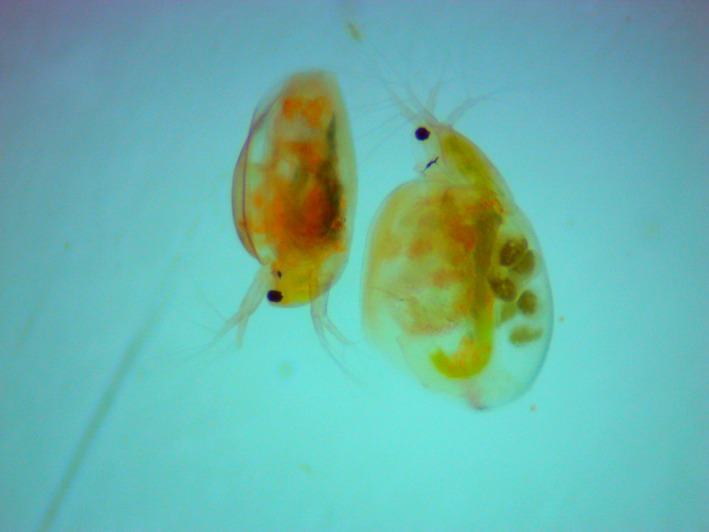
Example *Daphnia pulex* sampled from the CEZ

**Figure 2:**
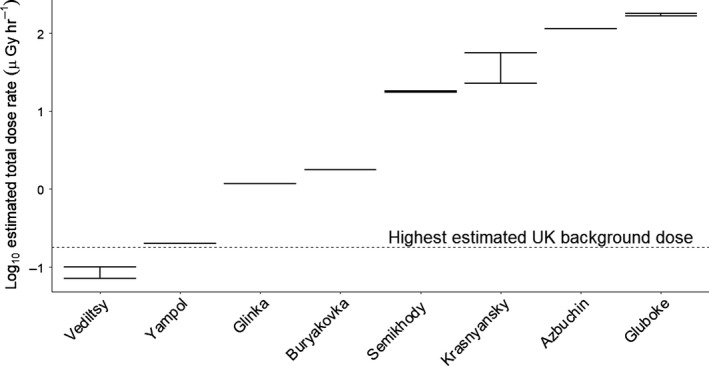
Log_10_ estimated total absorbed dose rates based on dose assessments made for each lake site, with ranges where appropriate (see Supporting Information Table S1 for the locations of each population). The black dotted line represents the highest estimated UK background dose rate of 0.18 µGy/h for comparison (Oatway, Jones, Holmes, Watson, & Cabianca, 2010; RIMNET, 2017)

We collected 38 *Daphnia* genotypes from the eight inhabited lake populations and maintained them as isofemale lines (henceforth called lines, Figure 1, see Supporting Information Table S2 for information on genotypes per lake). Each of the eight populations has experienced different levels of chronic radiation exposure (see Figure 2, Supporting Information Table S1). *Daphnia* samples were collected at one‐meter depths using a plankton net (net mesh: 0.25 mm, bag depth: 300 mm, outer frame: 250 mm diameter). The animals were transported to the laboratory in Chernobyl within three hours of sampling. Isofemale lines were then established by placing the *Daphnia* individually in 50 ml falcon tubes with water collected from the corresponding lake; these lines were allowed to propagate clonally. *Daphnia* lines were transferred to noncontaminated natural mineral water and fed *Chlorella vulgaris* algae for transport back to the laboratory at the University of Stirling (where the life history experiment took place). Once in Stirling, the *Daphnia* lines were maintained in a climate control facility under standard conditions without further exposure to radiation above UK natural background levels (20°C on a 12:12 hr light: dark cycle in 80 ml of artificial *Daphnia* media (ADaM, Klüttgen, Dülmer, Engels, & Ratte, 1994. Highest recorded UK natural background dose rate was 0.18 µGy/h in 2017 (RIMNET, 2017). We replaced the media and fed each genotype with 5 ml of *Chlorella vulgaris* three times weekly. Each line was maintained under standard conditions for three generations to minimize phenotypic variation due to maternal effects.

To assess radionuclide concentrations at each sample site, we extracted data, where available from the Ukraine atlas (Intelligence Systems GEO, 2008), for ^137^Cs and ^90^Sr (the dominant radionuclides in Chernobyl) and ^241^Am and ^239^Pu, which were considered representative of other radionuclides within the water column and upper sediment (IAEA, 2008). Where no data were available in the literature, sediment and water samples were taken at each sample site and transported to the Ukrainian HydroMeteorological Institute (UHMI) for analysis.

Water samples were analyzed as follows. First, 5–25 L of water was collected at each sample site and passed through an on‐line filtration system using a combination filter (Petryanov's FFP‐15‐1.5 prefilter + Blue Ribbon Grade paper filter) with a cartridge containing sorbent ANFEZH® to concentrate ^137^Cs and ^90^Sr. Following this, the cartridge was removed, and the filtered water was spiked with the radiochemical tracers ^243^Am and ^242^Pu and acidified to pH 2 with Nitric Acid followed by radiochemical separation. In the laboratory at the UHMI, the filter and sorbent were dried at 105°C to a constant weight, thoroughly mixed and packed in container for gamma spectrometry analysis. Where radioactivity levels were high enough, a subsample of water was taken for direct gamma measurement.

Sediment samples were taken as sediment cores, using a Kayak type sediment corer (made at the UHMI) from the deepest lake location (verified by echo‐sound measurements). Sediment core quality was assessed based upon two parameters, that there was no disturbance between the upper sediment along the core tube and that contrasting properties at the base of the core were present, indicating formation prior to the Chernobyl accident in 1986. In the UHMI laboratory, the sediment cores were sliced into sections (1–5 cm in size), freeze dried, homogenized, and submitted for gamma spectrometry analysis. Representative subsamples from selected slices (0.5–1.0 g) were taken for radiochemical analysis.

Radiometric analysis for ^137^Cs and ^241^Am was conducted using a gamma spectrometer with HPGe detector GMX‐40‐LB (Ortec, USA). ^90^Sr and transuranic elements (^238,239,240^Pu and ^241^Am) were preconcentrated using carbonate/hydroxides precipitation followed by serial extraction chromatography separation on Sr‐Resin and TRU‐Resin (Eichrom, USA) with ^90^Sr measured on a Liquid Scintillation Counter (TriCarb 2900TR; Perkin‐Elmer, USA), according to established methods (Laptev, Pirnach, & Dyvak, 2015) or alpha‐spectrometry on Alpha‐8 instrument (BSI, Latvia) after electrodeposition in the case of transuranic elements. Combined uncertainty of the ^137^Cs, ^90^Sr, and transuranic element activity measurements did not exceed 10%, 20%, and 30%, respectively.

The dose rate from internal and external radionuclides was estimated using ERICA (version 1.2), a software program designed to estimate radiation risk to wildlife based upon a range of representative species (Beresford et al., 2007; Brown et al.., 2008, 2016; ICRP, 2009). ERICA assessments were made by calculating dose rates based upon the activity concentrations provided and data on environmental radionuclide transfer. Our calculations were based on the default reference organism, Zooplankton, within ERICA. Zooplankton was selected on the basis of the geometry and size of *D. pulex* collected. Occupancy (which refers to the location of the organism within the lake) was changed to 75% surface sediment and 25% water column reflecting the fact the *Daphnia* population lies dormant throughout the autumn/winter as resting eggs in the surface sediment, before hatching in spring (Alekseev & Lampert, 2001), and that they vertically migrate throughout the water column (from sediment to water surface) to obtain food throughout the rest of the year (Dawidowicz & Loose,^1992^; McLaren, 1963). These occupancy rates should have produced a conservative estimate of the dose rate as the majority of the radionuclides were expected to have accumulated within the lake sediment (Nazarov & Gudkov, 2008). Activity concentrations are given in the Supporting Information Table S3.

### 2.3 Life history experiment

On day one of the experiment, *Daphnia* neonates were assigned to fresh jars and maintained under standard conditions. Offspring from the third clutch from the third generation of *Daphnia* were used as experimental replicates to minimize variation due to maternal effects. Where maternal lines did not produce their third clutch from the third generation of *Daphnia* on day one of the experiment, the neonates were assigned to fresh jars thereafter, and the experimental days were standardized for statistical analysis. We measured the fecundity and survival of females daily from each of the 30 *Daphnia* isofemale lines from eight lake populations that had experienced different historical radiation doses. Fecundity was recorded as the day of each brood release and the number of offspring produced in each brood. Survival was measured by recording the day of death for each individual. There were eight replicates per line, where each replicate consisted of a single *Daphnia* in 50 ml of artificial *Daphnia* medium (ADaM; see Klüttgen et al., 1994). Replicate animals were fed 1.0 ABS *Chlorella vulgaris* algal cells per day (where ABS is the optical absorbance 650 nm white light) and the media was replaced when offspring clutches were released.

### 2.4 Statistical analysis

Analyses were performed using R statistical software (R Core Team, 2017) version 3.4.3. First, we tested the effects of dose rate and lake population on *Daphnia *survival. Specifically, we fitted mixed effects Cox's proportional Hazards (CoxME) models to the survival data using the *coxme *package (Therneau, 2015, 2018), where dose rate was fitted as a covariate and lake population was fitted as a fixed effect. Line nested within lake population was included as a random effect to account for the fact that we measured multiple genotypes per lake. Significant effects of lake population were further investigated using a *post hoc* Tukey test to determine which populations were different from each other using the *multcomp* package (Hothorn et al.,^2008^).

The effects of dose rate and lake population on the total number of offspring produced were tested using generalized linear mixed models with Poisson error distribution (GLMM, implemented the *lme4* package; Bates, Machler, Bolker, & Walker, 2015), where line within lake population was included as a random effect. Significant differences identified between lake populations were tested using a Tukey's range post hoc test. Using the same approach and random effects structure, but with a binomial distribution (as individuals were either identified as reproducing or not reproducing), we tested whether the number of non‐reproducing individuals varied according to dose rate or lake population. Next, we examined how dose rate and lake population affected age‐specific reproduction using generalized additive mixed models (GAMMs within the *gamm4* package; Wood & Scheipl, 2017). GAMMs are semiparametric models that are useful for predicting nonlinear effects, where the linear predictor is dependent on a “smooth” function, which determines the level of smoothness in the fitted curve. This smooth function can depend on one or multiple nonparametric smoothers fitted to factors or covariates. We compared a model where smoothers were fitted to both experimental day and either dose rate or lake population to a model where a smoother was fitted to experimental day only. Random effects included replicate nested within line nested within lake population, to account for the fact that repeated fecundity measures were taken for each individual. In addition, we made pairwise comparisons of smoothed and unsmoothed models for combinations of pooled lake populations. The best fit model was determined using Akaike's information criterion (AIC), where the model with the lowest AIC was considered the best model and models with an AIC difference of less than two were regarded as the same (Burnham & Anderson, 2002).

Finally, we assessed overall population fitness by calculating the instantaneous rate of population increase (*r*) for each genotype using the Euler–Lotka equation:

$$1=\sum_{x=0}^{n}e^{-rx}l_{x}m_{x},$$

Where *x* represents the age of each organism in days, *l_x_* is the proportion of surviving females at each age classification, and *m_x_* is the number of offspring produced at each corresponding age (Birch, 1948; Cuco, Castro, Gonçalves, Wolinska, & Abrantes, 2017; Grant & Grant, 1992). We tested for variation in *r* across lake populations and by dose rate using generalized least squares models (GLS models using the *nlme* package) (Pinheiro, Bates, DebRoy, & Sarkar, 2018), where the intercept was allowed to vary by lake population. We tested for normality of distribution of *r* data using the Shapiro–Wilk and then performed a Bartlett's test to determine if variances in *r* differed according to lake population. Where dose rates were not normally distributed, a Fligner‐Killeen test was performed to test if variance in *r* is associated with dose rate.

## 3 RESULTS

### 3.1 Radiation exposure does not affect Daphnia survival

**Figure 3:**
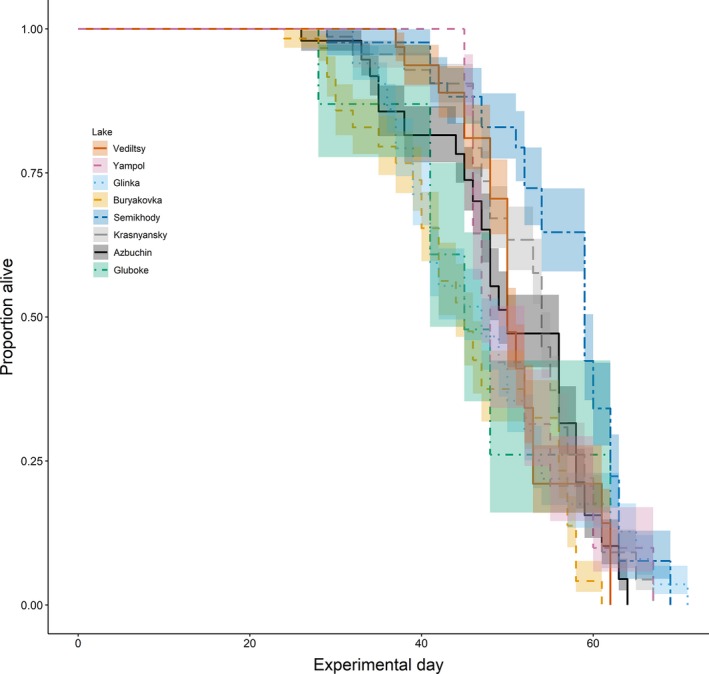
Variation in *Daphnia* survival according to lake population (shaded regions denote ±95% confidence intervals [CIs])

We found no effect of dose rate on *Daphnia* survival (CoxME: coefficient = −0.001 ± 0.004, *z* = 0.15, *p* = 0.88). There were significant differences in survival across lake populations (CoxME: $\chi_{7}^{2}$ = 920.73, *p* < 0.0001, Figure 3. Median day of death in Vediltsy: 50, Yampol: 48, Glinka: 47, Buryakovka: 45, Semikhody: 59, Krasnyansky: 54, Azbuchin: 50, Gluboke: 45).

### 3.2 Radiation exposure does not affect reproduction

**Figure 4:**
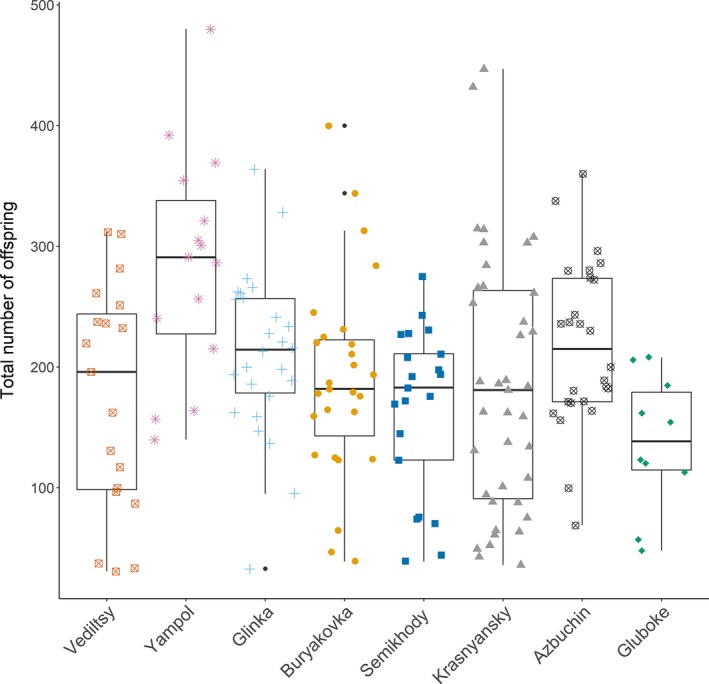
Boxplot showing the total number of offspring produced by each lake population. Populations are plotted in order of increasing dose rate. The box shows the upper and lower quartiles within the data and the line within each box shows the median value. The lines outside of each box show the range of the data

There was a significant effect of dose rate (GLMM: $\chi_{1}^{2}$ = 64.89, *p* < 0.0001) and lake population (GLMM: $\chi_{7}^{2}$ = 995.99, *p* < 0.0001) on the total number of offspring produced, though the variation in total offspring was better explained by population (∆AIC = 981.99). Tukey's post hoc test revealed that in all cases, this variation was driven entirely by lake Yampol (categorized as very low, *p* < 0.05 for comparisons between Yampol and all other lake populations) (see Figure 4). The proportion of non‐reproducing *Daphnia *varied between 0.125 and 0.658 across lines. Analysis found a marginally non‐significant effect of dose rate on the likelihood of individual failure to reproduce (GLMM: $\chi_{1}^{2}$ = −3.6, *p* = 0.06); this suggests that if radiation‐induced sterility does occur, it is unlikely to have a strong effect on population‐level fecundity. By contrast, there were significant differences in the proportion of non‐reproducing individuals among lakes (GLMM: $\chi_{8}^{2}$ = −31.67, *p* < 0.001). *Post* *hoc* testing revealed that this was driven by a high incidence of non‐reproducers in Yampol lake (*p* < 0.05, see Table S2).

**Figure 5:**
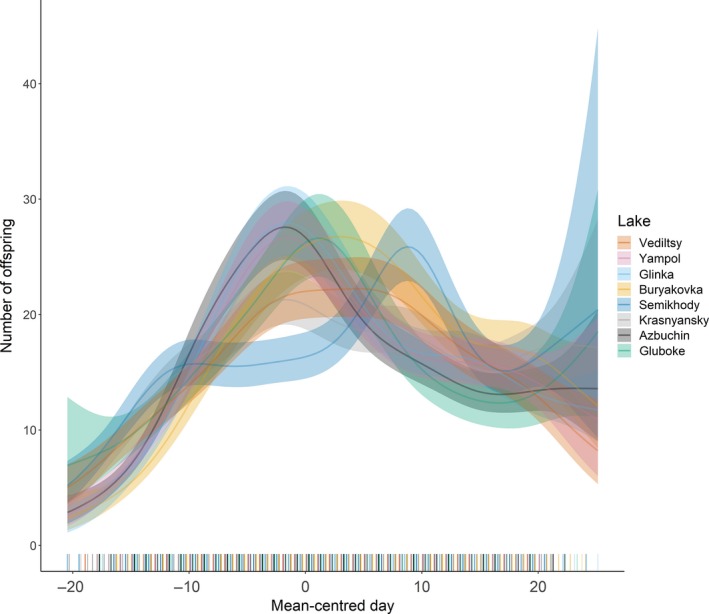
Age‐specific reproduction according to Lake Population. The lines represent predictions based on a Generalized Additive Mixed Model (GAMM) fitting the number of offspring produced on each mean‐centered day, smoothed by lake population. Replicate nested within line nested within lake was fitted as a random effect. The shaded areas show 95% confidence intervals (CIs). The model was fitted using the *visreg* package (Breheny & Burchett, 2017)

Comparisons between models revealed that lake population explained more variation in age‐specific reproduction than dose rate (see Table 1). Further, smoothing the day of reproduction by lake population significantly improved the model fit compared to fitting lake population as a parametric fixed effect (GAMM: ∆AIC = 482.18, $\chi_{14}^{2}$ = 510.17, *p* < 0.0001). The best fitting model included day by lake population as a nonparametric smoother and showed that all lakes varied from one another (Table 2) and that the timing of reproductive peaks varies across populations (Figure 5).

**Table 1:** Summary for Generalized Additive Mixed Models (GAMMs) assessing age‐specific reproduction

**Response**	**Parametric/smoother**	**Term**	**AIC**
Offspring production	Parametric	Dose rate	15,635.83
	Smoother	Dose rate	17,437.41
	Parametric	Lake population	15,635.83
	Smoother	Lake population	15,153.65

Note

In all models, replicate nested within line nested within lake is fitted as a random effect. *n* = 1,899.

**Table 2:** Generalized Additive Mixed Model (GAMM) fitting age‐specific reproduction data by lake population

**Response**	**Parametric/smoother**	**Term**	***df*** **(eDF)**	**χ^2^**	***p***
Offspring production	Smoother	Day by Buryakovka	6.27	543.1	**<0.0001**
	Smoother	Day by Yampol	6.11	463.4	**<0.0001**
	Smoother	Day by Vediltsy	5.63	209.0	**<0.0001**
	Smoother	Day by Glinka	6.78	607.3	**<0.0001**
	Smoother	Day by Semikhody	7.28	221.6	**<0.0001**
	Smoother	Day by Krasnyansky	7.08	382.4	**<0.0001**
	Smoother	Day by Azbuchin	6.88	693.5	**<0.0001**
	Smoother	Day by Gluboke	5.46	127.6	**<0.0001**

Note

Day by lake population is fitted as a nonparametric smoother and replicate nested within line nested within lake is fitted as a random effect. eDF is the estimated degrees of freedom. *N* = 1,899.

### 3.3 Radiation exposure does not affect overall fitness

**Figure 6:**
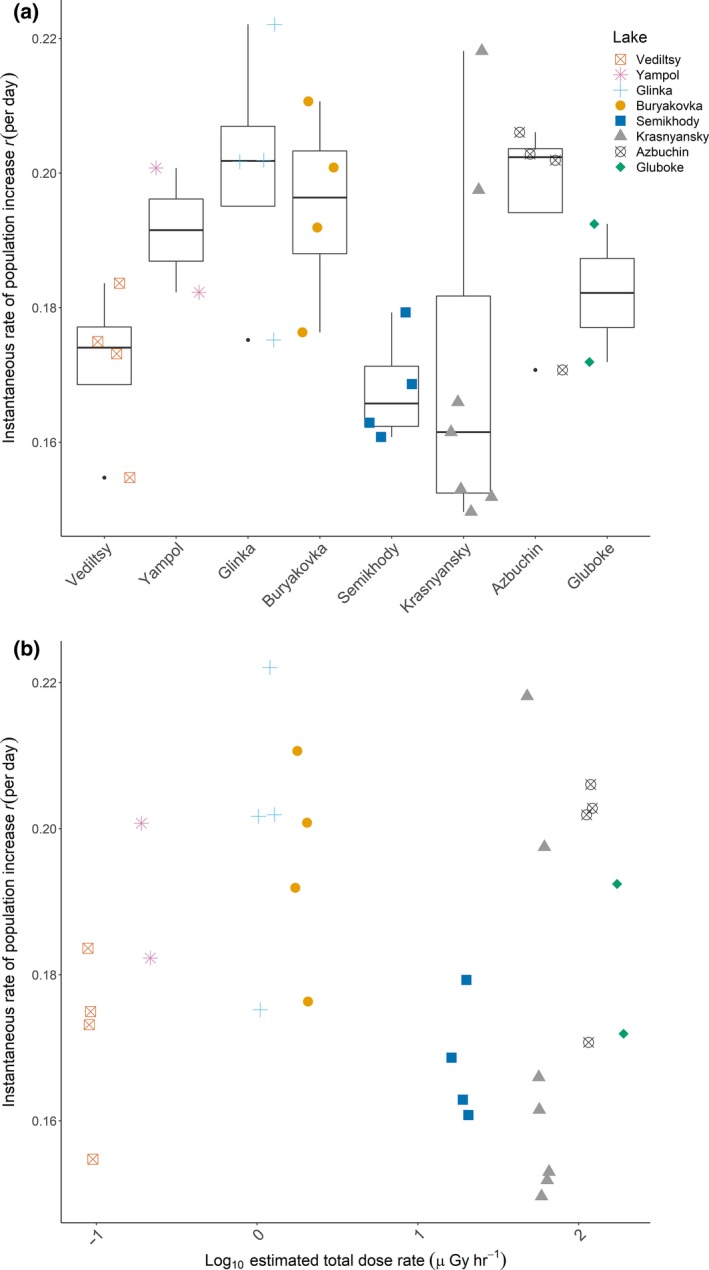
Instantaneous rates of population increase *r* (day^‐1^) for each genotype (a) according to dose rate and (b) by lake population. Populations are plotted in order of increasing dose rate. The box shows the upper and lower quartiles within the data and the line within each box shows the median value. The lines outside of each box show the range of the data

We found no effect of dose rate (GLS: *F*_1,29_ = 0.001, *p* = 0.98, Figure 6a) or lake population (GLS: *F*_7,23_ = 2.08, *p* = 0.09; Figure 6b) on *r*. Variation in *r *did not vary according to dose rate ($\chi_{7}^{2}$ = 2.58, *p* = 0.92, Figure 6a) or lake population (Bartlett's $K_{7}^{2}$ = 4.97, *p* = 0.66, Figure 6b).

## 4 DISCUSSION

In this study, we presented the results of an experiment designed to examine variation in *Daphnia* survival and fecundity across populations in Chernobyl that have experienced very different levels of exposure to chronic radiation. We found no overall effect of dose rate on *Daphnia* survival. Laboratory‐based studies have previously demonstrated that ionizing radiation negatively affects invertebrate (including *Daphnia*) survival at higher levels than those tested within the present study (Nohara et al., 2014; Parisot et al., 2015; Sarapultseva & Gorski, 2013). Parisot et al., (2015) found elevated mortality in *Daphnia* under radiation exposure, but only when animals were exposed for multiple generations under very high dose rates (4.7 × 10^3^ µGy/h and 3.54 × 10^4^ µGy/h); these are much higher doses than those found in the CEZ, (we estimated ~180 µGy/h in Gluboke lake, which experienced the highest dose rate). However, this is not to say that exposure to radiation cannot affect natural populations; for example, CEZ populations have been exposed over a considerably longer period and to a variety of additional stressors that may have confounding impacts (Holmstrup et al., 2010).

After a careful and detailed examination of *Daphnia* reproduction—from total offspring output to subtle changes in reproductive investment through age‐specific reproduction and proportion of non‐reproducing individuals— we found no evidence for radiation‐mediated effects. Variability in total offspring output was driven by lake Yampol (categorized as a very low dose rate) only and each lake population had a unique pattern of offspring production with variable timing of peak reproduction, independent of dose rate. There is limited research on radiation‐mediated life history shifts in wild populations, and these studies found that irradiated groups invested in greater reproductive output but had similar overall population sizes due to differences in survival or reproductive schedules (Blaylock, 1969; Cooley, 1973). The fact we find no effect of dose rate on *Daphnia *survival may explain why we observe no correlated effect on reproduction.

Whilst reproduction and survival provide valuable measures of fitness, the timing of reproductive investment with respect to lifespan is also important. The instantaneous rate of population increase (*r*) is a particularly useful measure, because it accounts for the fact that offspring produced in early life make a greater contribution to the mother's fitness than those produced later (Birch, 1948). We calculated *r *for each isofemale line and determined if mean or variance in *r *varied according to radiation dose rate. Specifically, we tested whether: (a) *r* declines and variation in *r* increases with dose rate, consistent with radiation‐mediated supply of mutations reducing overall fitness whilst increasing variation; or (b) that there would be no overall difference in mean *r* across populations, but variation in *r* would decline with increasing dose rate, consistent with stronger selection at higher radiation levels. Radiation dose rate was not associated with either the mean or variance in *r, *showing that historic radiation exposure is not the primary driver of variation in *Daphnia *fitness in these Chernobyl populations.

It is important to acknowledge that lack of association between dose rate and life history variation at the population level does not mean that radiation is not having any effect. Radiation‐mediated effects on reproduction within individual *Daphnia* have been demonstrated in the laboratory at dose rates as low as 7 µGy/h (Parisot et al., 2015). However, in natural populations, a variety of ecological factors such as competition, predation or parasitism apply strong and often variable selection on populations (Auld et al., 2013; Brockelman, 1975; Creel & Christianson, 2008; Lehmann, 1993). These ecological factors are therefore likely to be bigger drivers of life history variation than current dose rates. This brings into sharp focus the fact that few studies consider how the effects of radiation on individuals might scale to effects at the population or ecosystem level. A notable exception is a conceptual model by Polikarpov that predicts the negative effects of radiation on individuals will be overshadowed by much stronger interactions between the population and the wider ecosystem at higher radiation doses (termed "ecological masking"; Polikarpov, 1998). Notably, the estimated dose rates in this study (~0.10 – 180 µGy/h) fall within those predicted to cause the “Ecological masking zone” in Polikarpov's model.

We tested whether key life history traits varied across *Daphnia *populations that experienced a wide range of chronic radiation exposure in the Chernobyl Exclusion Zone. We found no such effects. It is clear that although radiation is known to negatively affect individuals, we need to view it as one of many sources of selection in ecologically complex communities. Future research needs to widen the focus to other highly contaminated areas such as Fukushima (Saito et al., 2015) and dissect the possible interactions between radiation and other stressors on individual fitness. The challenge now is to quantify the impacts of radiation relative to competition, predation, parasitism *etc. *in order to have a more complete understanding of the effects on radiation on the wider ecosystem.

## CONFLICT OF INTEREST

None declared.

## AUTHOR CONTRIBUTION

The experiment was designed by JG, SKJRA, and DC. JG and SG performed the field work. GVL performed radiometric analysis of water and sediment samples. JG conducted the experiment. JG and SKJRA conducted the statistical analysis. JG, SKJRA, and DC wrote the manuscript. SG and GVL provided useful comments on the manuscript. All authors read and approved the final version of the manuscript.

## Data Availability

Data can be accessed on the Dryad repository (https://doi.org/10.5061/dryad.jr412dq).

## References

[ece34931-bib-0001] Alekseev, V. , & Lampert, W. (2001). Maternal control of resting‐egg production in Daphnia. Nature, 414, 899–901. 10.1038/414899a 11780060

[ece34931-bib-0002] Auld, S. K. J. R. , & Brand, J. (2017). Simulated climate change, epidemic size, and host evolution across host–parasite populations. Global Change Biology, 23, 5045–5053. 10.1111/gcb.13769 28544153

[ece34931-bib-0003] Auld, S. K. J. R. , Penczykowski, R. M. , Ochs, J. H. , Grippi, D. C. , Hall, S. R. , & Duffy, M. A. (2013). Variation in costs of parasite resistance among natural host populations. Journal of Evolutionary Biology, 26, 2479–2486. 10.1111/jeb.12243 24118613

[ece34931-bib-0004] Baker, R. J. , Hamilton, M. J. , Van Den Bussche, R. A. , Wiggins, L. E. , Sugg, D. W. , Smith, M. H. , … Chesser, R. K. (1996). Small mammals from the most radioactive sites near the Chornobyl nuclear power plant. Journal of Mammalogy, 77, 155–170. 10.2307/1382717

[ece34931-bib-0005] Ball, S. L. , & Baker, R. L. (1996). Predator‐induced life history changes: Antipredator behavior costs or facultative life history shifts? Ecology, 77, 1116–1124. 10.2307/2265580

[ece34931-bib-0006] Bates, D. , Machler, M. , Bolker, B. , & Walker, S. (2015). Fitting linear mixed‐effects models using lme4. Journal of Statistical Software, 67, 1–48.

[ece34931-bib-0007] Beresford, N. , Brown, J. , Copplestone, D. , Garnier‐, J. , Howard, B. , & Larsson, C.‐M. … Zinger, I. (2007) *D‐ERICA: an integrated approach to the assessment and management of environmental risks from ionising radiation* . Descr. Purp. Methodol. Appl. EC Proj. Contract no. FI6R‐CT‐2004‐508847. Retrieved from www.erica‐project.org.

[ece34931-bib-0008] Bickham, J. W. , Sandhu, S. , Hebert, P. D. N. , Chikhi, L. , & Athwal, R. (2000). Effects of chemical contaminants on genetic diversity in natural populations: Implications for biomonitoring and ecotoxicology. Mutation Research, 263, 33–51. 10.1016/S1383-5742(00)00004-1 10838208

[ece34931-bib-0009] Bickham, J. W. , & Smolen, M. J. (1994). Somatic and heritable effects of environmental genotoxins and the emergence of evolutionary toxicology. Environmental Health Perspectives, 102, 25–28. 10.1289/ehp.94102s1225 PMC1566727 7713028

[ece34931-bib-0010] Birch, L. C. (1948). The intrinsic rate of natural increase of an insect population. Journal of Animal Ecology, 17, 15–26. 10.2307/1605

[ece34931-bib-0011] Blaylock, B. (1969). The Fecundity of a *Gambusia affinis* population exposed to chronic environmental radiation. Radiation Research, 37, 108–117. 10.2307/3572756 5762913

[ece34931-bib-0012] Bréchignac, F. (2017). Assessing ecological risk from radiation requires an ecosystem approach In Korogodina V.L., Mothersill C.E., Inge-Vechtomov S.G., & C.B Seymour (Eds.), Genetics, Evolution and Radiation (pp. 207–223). the Netherlands: Springer.

[ece34931-bib-0013] Breheny, P. , & Burchett, W. (2017). Visualization of regression models using visreg. The R Journal, 9, 56–71.

[ece34931-bib-0014] Breimer, L. H. (1988). Ionizing radiation‐induced mutagenesis. British Journal of Cancer, 57, 6–18. 10.1038/bjc.1988.2 3279995 PMC2246688

[ece34931-bib-0015] Brockelman, W. Y. (1975). Competition, the fitness of offspring, and optimal clutch size. American Naturalist, 109, 677–699. 10.1086/283037

[ece34931-bib-0016] Brown, J. E. , Alfonso, B. , Avila, R. , Beresford, N. A. , Copplestone, D. , & Hosseini, A. (2016) A new version of the ERICA tool to facilitate impact assessments of radioactivity on wild plants and animals. Journal of Environmental Radioactivity, 153: 141–148. 10.1016/j.jenvrad.2015.12.011 26773508

[ece34931-bib-0017] Brown, J. E. , Alfonso, B. , Avila, R. , Beresford, N. A. , Copplestone, D. , Pröhl, G. , & Ulanovsky, A. (2008). The ERICA tool. Journal of Environmental Radioactivity, 99, 1371–1383. 10.1016/j.jenvrad.2008.01.008 18329765

[ece34931-bib-0018] Burnham, K. P. , & Anderson, D. R. (2002). Model selection and multimodel inference: A practical information‐theoretic‐approach (2nd ed.). New York, NY, London, UK: Springer.

[ece34931-bib-0019] Cooley, J. (1973) Effects of chronic environmental radiation on a natural population of the aquatic snail *Physa heterostropha* . Radiation Research, 54: 130–140. 10.2307/3573871 4699790

[ece34931-bib-0020] Creel, S. , & Christianson, D. (2008). Relationships between direct predation and risk effects. Trends in Ecology & Evolution, 23, 194–201. 10.1016/j.tree.2007.12.004 18308423

[ece34931-bib-0021] Crow J.F. (1970). Genetic Loads and the Cost of Natural Selection In: Kojima K. (Ed.), Mathematical Topics in Population Genetics. Biomathematics (pp 32–78), Berlin, Heidelberg: Springer.

[ece34931-bib-0022] Cuco, A. P. , Castro, B. B. , Gonçalves, F. , Wolinska, J. , & Abrantes, N. (2017). Temperature modulates the interaction between fungicide pollution and disease: Evidence from a Daphnia‐microparasitic yeast model. Parasitology, 145:939‐947. 29160185 10.1017/S0031182017002062

[ece34931-bib-0023] Dawidowicz, P. , & Loose, C. J. (1992). Cost of swimming by daphnia during diel vertical migration. Limnology and Oceanography, 37, 665–669.

[ece34931-bib-0024] Decaestecker, E. , De Meester, L. , & Mergeay, J. (2009). Cyclical parthenogenesis in daphnia: Sexual versus asexual reproduction In Schön I., Martens K., & vanDijk P. (Eds.), Lost sex: The evolutionary biology of parthenogenesis (pp. 295–316). Amsterdam, the Netherlands: Springer Netherlands.

[ece34931-bib-0025] Esnault, M.‐A. , Legue, F. , & Chenal, C. (2010). Ionizing radiation: Advances in plant response. Environmental and Experimental Botany, 68, 231–237. 10.1016/j.envexpbot.2010.01.007

[ece34931-bib-0026] Flaherty, C. M. , & Dodson, S. I. (2005). Effects of pharmaceuticals on Daphnia survival, growth, and reproduction. Chemosphere, 61, 200–207. 10.1016/j.chemosphere.2005.02.016 16168743

[ece34931-bib-0027] Galván, I. , Bonisoli‐Alquati, A. , Jenkinson, S. , Ghanem, G. , Wakamatsu, K. , Mousseau, T. A. , & Møller, A. P. (2014). Chronic exposure to low‐dose radiation at Chernobyl favours adaptation to oxidative stress in birds. Functional Ecology, 28, 1387–1403. 10.1111/1365-2435.12283

[ece34931-bib-0028] Grant, P. R. , & Grant, R. B. (1992). Demography and the genetically effective sizes of two populations of Darwin’s finches. Ecology, 73, 766–784.

[ece34931-bib-0029] Haag, C. R. , Riek, M. , Hottinger, J. W. , Pajunen, V. I. , & Ebert, D. (2006). Founder events as determinants of within‐island and among‐island genetic structure of Daphnia metapopulations. Heredity, 96, 150–158. 10.1038/sj.hdy.6800774 16369578

[ece34931-bib-0030] Haldane, J. B. S. (1937). The effect of variation on fitness. American Naturalist, 71, 337–349. 10.1086/280722

[ece34931-bib-0031] Hinton, T. G. , Alexakhin, R. , Balonov, M. , Gentner, N. , Hendry, J. , Prister, B. , … Woodhead, D. (2007). Radiation‐induced effects on plants and animals : Findings of the United Nations Chernobyl Forum. Health Physics, 93, 427–440. 10.1097/01.HP.0000281179.03443.2e 18049219

[ece34931-bib-0032] Holmstrup, M. , Bindesbøl, A. M. , Oostingh, G. J. , Duschl, A. , Scheil, V. , Köhler, H. R. … Spurgeon, D. (2010). Interactions between effects of environmental chemicals and natural stressors: A review. The Science of the Total Environment, 408, 3746–3762. 10.1016/j.scitotenv.2009.10.067 19922980

[ece34931-bib-0033] Hothorn, T. , Bretz, F. , & Westfall, P. (2008). Simultaneous inference in general parametric models. Biometrical Journal, 50, 346–363. 18481363 10.1002/bimj.200810425

[ece34931-bib-0034] Husseneder, C. , Donaldson, J. R. , & Foil, L. D. (2016). Impact of the 2010 Deepwater Horizon oil spill on population size and genetic structure of horse flies in Louisiana marshes. Scientific Reports, 6, 18968 10.1038/srep18968 26755069 PMC4709594

[ece34931-bib-0035] IAEA (2008). Annual Report 2008. Retrieved from https://www.iaea.org/sites/default/files/publications/reports/2008/anrep2008_full.pdf (accessed 14.01.19.).

[ece34931-bib-0036] ICRP (2009). *Transfer parameters for reference animals and plants* . Ann. ICRP 39. 10.1016/j.icrp.2011.08.009 22108188

[ece34931-bib-0037] Intelligence Systems GEO, L . (2008). *Atlas Ukraine radioactive contamination*. order Minist.Ukr. Emergencies Aff. Popul. Prot. from Consequences Chernobyl Catastr.

[ece34931-bib-0038] International Atomic Energy Agency (2006). *Environmental consequences of the Chernobyl accident and their remediation: Twenty years of experience*. IAEA , Vienna 167.

[ece34931-bib-0039] Kimura, M. , & Maruyama, T. (1966). The mutational load with epistatic gene interactions in fitness. Genetics, 54, 1337–1351. 17248359 10.1093/genetics/54.6.1337 PMC1211299

[ece34931-bib-0040] Klüttgen, B. , Dülmer, U. , Engels, M. , & Ratte, H. (1994). ADaM, an artificial freshwater for the culture of zooplankton. Water Research, 28, 743–746. 10.1016/0043-1354(94)90157-0

[ece34931-bib-0041] Kryshev, I. I. (1995). Radioactive contamination of aquatic ecosystems following the Chernobyl accident. Journal of Environmental Radioactivity, 27, 207–219. 10.1016/0265-931X(94)00042-U

[ece34931-bib-0042] Laptev, G. V. , Pirnach, L. S. , & Dyvak, T. I. (2015). Determination of 90Sr in water by direct measurement using liquid scintillation counter. Nuclear Physics and Atomic Energy, 16, 177–182.

[ece34931-bib-0043] Lehmann, T. (1993). Ectoparasites: Direct impact on host fitness. Parasitology Today, 9, 8‐13. 15463655 10.1016/0169-4758(93)90153-7

[ece34931-bib-0044] McLaren, I. A. (1963). Effects of temperature on growth of zooplankton, and the adaptive value of vertical migration. Journal of the Fisheries Research Board of Canada, 20, 685–727. 10.1139/f63-046

[ece34931-bib-0045] McLaughlin, J. F. , Hellmann, J. J. , Boggs, C. L. , & Ehrlich, P. R. (2002). Climate change hastens population extinctions. Proceedings of the National Academy of Sciences of the United States of America, 99, 6070–6074. 10.1073/pnas.052131199 11972020 PMC122903

[ece34931-bib-0046] Morgan, W. F. (2003a). Non‐targeted and delayed effects of exposure to ionizing radiation: I. Radiation‐induced genomic instability and bystander effects in vitro. Radiation Research, 159, 567–581. 10.1667/0033-7587(2003)159[0567:NADEOE]2.0.CO;2 12710868

[ece34931-bib-0047] Morgan, W. F. (2003b). Non‐targeted and delayed effects of exposure to ionizing radiation: II. Radiation‐induced genomic instability and bystander effects in vitro. Radiation Research, 159, 581–596. 10.1667/0033-7587(2003)159[0581:NADEOE]2.0.CO;2 12710869

[ece34931-bib-0048] Murphy, J. F. , Nagorskaya, L. L. , & Smith, J. T. (2011). Abundance and diversity of aquatic macroinvertebrate communities in lakes exposed to Chernobyl‐derived ionising radiation. Journal of Environmental Radioactivity, 102, 688–694. 10.1016/j.jenvrad.2011.04.007 21530025

[ece34931-bib-0049] Nazarov, A. , & Gudkov, D. (2008). *Radiation monitoring of lake ecosystems within the Chernobyl accident exclusion zone* .

[ece34931-bib-0050] NEA (2002) *The release, dispersion and deposition of radionuclides* . In: Chernobyl: Assessment of Radiological and Health Impact 2002 Update of Chernobyl: Ten Years On.

[ece34931-bib-0051] Nohara, C. , Taira, W. , Hiyama, A. , Tanahara, A. , Takatsuji, T. , & Otaki, J. M. (2014). Ingestion of radioactively contaminated diets for two generations in the pale grass blue butterfly. BMC Evolutionary Biology, 14, 193 10.1186/s12862-014-0193-0 25330067 PMC4171559

[ece34931-bib-0052] Oatway, W. B. , Jones, A. L. , Holmes, S. , Watson, S. , & Cabianca, T. (2010). *Ionising radiation exposure of the UK population: 2010 review* .

[ece34931-bib-0053] OECD (2012). Test No. 211: Daphnia magna reproduction test. Paris, France: OECD Publishing.

[ece34931-bib-0054] Pace, M. L. , Cole, J. J. , Carpenter, S. R. , Kitchell, J. F. , Hodgson, J. R. , Van De Bogert, M. C. , … Bastviken, D. (2004). Whole‐lake carbon‐13 additions reveal terrestrial support of aquatic food webs. Nature, 427, 240–243. 10.1038/nature02227 14724637

[ece34931-bib-0055] Parisot, F. , Bourdineaud, J.‐P. , Plaire, D. , Adam‐Guillermin, C. , & Alonzo, F. (2015). DNA alterations and effects on growth and reproduction in Daphnia magna during chronic exposure to gamma radiation over three successive generations. Aquatic Toxicology, 163, 27–36. 10.1016/j.aquatox.2015.03.002 25840277

[ece34931-bib-0056] Pinheiro, J. , Bates, D. , DebRoy, S. , Sarkar, D. , & R Core Team. (2018). *Linear and nonlinear mixed effects models* .

[ece34931-bib-0057] Polikarpov, G. (1998). Conceptual model of responses of organisms, populations and ecosystems to all possible dose rates of ionising radiation in the environment. Radiation Protection Dosimetry, 75, 181–185. 10.1093/oxfordjournals.rpd.a032225

[ece34931-bib-0058] R Core Team (2017). R: A Language and Environment for Statistical Computing. Vienna, Austria: R Core Team.

[ece34931-bib-0059] Reznick, D. A. , Bryga, H. , & Endler, J. A. (1990). Experimentally induced life‐history evolution in a natural population. Nature, 346, 357–359. 10.1038/346357a0

[ece34931-bib-0060] Riffaut, L. , McCoy, K. D. , Tirard, C. , Friesen, V. L. , & Boulinier, T. (2005). Population genetics of the common guillemot Uria aalge in the North Atlantic: Geographic impact of oil spills. Marine Ecology Progress Series, 291, 263–273. 10.3354/meps291263

[ece34931-bib-0061] Saito, K. , Tanihata, I. , Fujiwara, M. , Saito, T. , Shimoura, S. , Otsuka, T. , … Shibata, T. (2015). Detailed deposition density maps constructed by large‐scale soil sampling for gamma‐ray emitting radioactive nuclides from the Fukushima Dai‐ichi Nuclear Power Plant accident. Journal of Environmental Radioactivity, 139, 308–319. 10.1016/j.jenvrad.2014.02.014 24703526

[ece34931-bib-0062] Santos, E. D. , Hamilton, P. B. , Coe, T. S. , Ball, J. S. , Cook, A. C. , Katsiadaki, I. , & Tyler, C. R. (2013). Population bottlenecks, genetic diversity and breeding ability of the three‐spined stickleback (Gasterosteus aculeatus) from three polluted English Rivers. Aquatic Toxicology, 142–143, 264–271. 10.1016/j.aquatox.2013.08.008 24071362

[ece34931-bib-0063] Sarapultseva, E. I. , & Gorski, A. I. (2013). Low‐dose γ‐Irradiation affects the survival of exposed daphnia and their offspring. Dose‐Response, 11, 460–468. 24298224 10.2203/dose-response.12-033.Sarapultseva PMC3834740

[ece34931-bib-0064] Saxen, R. , Taipale, T. K. , & Aaltonen, H. (1987). *Radioactivity of wet and dry deposition and soil in Finland after the Chernobyl accident in 1986 Supplement 2 to Annual Report STUK A55* .

[ece34931-bib-0065] The Radiation Incident Monitoring Network (RIMNET) (2017). *Ambient gamma radiation dose rates across the UK – GOV.UK* .

[ece34931-bib-0066] Therneau, T. (2015). *A package for survival analysis in R* .

[ece34931-bib-0067] Therneau, T. (2018). *coxme: Mixed effects cox models* .

[ece34931-bib-0068] UNSCEAR (2008). *Sources and Effects of Ionizing Radiation: Sources* . United Nations Sci. Comm. Eff. At. Radiat..

[ece34931-bib-0069] von Sonntag, C. (2007). DNA lesions induced by ionizing radiation In Vijayalaxmi O. G. (Ed.), Chromosomal alterations. Berlin, Heidelberg: Springer.

[ece34931-bib-0070] Wood, S. , & Scheipl, F. (2017). *gamm4: Generalized additive mixed models using “mgcv” and “lme4”* . CRAN.

[ece34931-bib-0071] Zaffagnini, F. (1987). Reproduction in Daphnia. Memorie Dell'istituto Italiano Di Idrobiologia, 45, 245–284.

