# Supplementary material for: Variation in chronic radiation exposure does not drive life history divergence among Daphnia populations across the Chernobyl Exclusion Zone
Source: Ecol Evol. 2019 Feb 3;9(5):2640–50. doi: 10.1002/ece3.4931 (PMC6405491; doi:10.1002/ece3.4931)
Supplement: Supplementary file 1 [file ECE3-9-2640-s001.docx]

**Supplementary information**

| **Table S1:** The names of sampling sites, coordinates and estimated radiation contamination levels. Coordinates are given using the Universal Transverse Mercator (UTM) system using the WGS84 ellipsoid. | | | | |
| --- | --- | --- | --- | --- |
| **Lake name** | **Co-ord N** | **Co-ord E** | **Total dose rate estimate (µGy h^-1^)** | **Radiation level** |
| Vediltsy | 51.4352 | 30.83846 | 0.10* | Very low |
| Yampol | 51.2095 | 30.17667 | 0.20* | Very low |
| Glinka | 51.2174 | 29.93713 | 1.20* | Low |
| Buryakovka | 51.3978 | 29.8931 | 1.80* | Low |
| Semikhody | 51.4151 | 30.0502 | 17.50 – 18.00 | Medium |
| Krasnyansky | 51.4429 | 30.07643 | 22.80 – 55.80 | Medium |
| Azbuchin | 51.408 | 30.11102 | 115.60 – 115.70 | High |
| Gluboke | 51.4454 | 30.06528 | 166.90 - 181.20 | High |
| *This is based on available data where in some cases calculation of range was not possible | | | | |

**Table S2**: Number of genotypes setup for each of the eight lake populations, number of non-reproducing individuals and the number of genotypes assessed in the experiment.

| **Lake population** | **Number of genotypes setup** | **Number of non-reproducing individuals** | **Proportion of non-reproducing individuals** | **Number of genotypes in the experiment** |
| --- | --- | --- | --- | --- |
| Vediltsy | 4 | 10 | 0.25 | 4 |
| Yampol | 6 | 31 | 0.658 | 2 |
| Glinka | 4 | 5 | 0.125 | 4 |
| Buryakovka | 5 | 11 | 0.275 | 4 |
| Semikhody | 4 | 21 | 0.525 | 4 |
| Krasnyansky | 7 | 14 | 0.25 | 6 |
| Azbuchin | 5 | 14 | 0.35 | 4 |
| Gluboke | 3 | 10 | 0.417 | 2 |
| *Total* | 38 |  |  | 30 |

| **Table S3**: ^137^Cs, ^90^Sr, ^241^Am and ^239^Pu activity concentrations in water and sediment samples collected from each lake site. Water (w) concentrations are in Bq l^-1^ and sediment (s) concentrations in Bq g^-1^ (dry weight). | | | | | | | | | | | | | |
| --- | --- | --- | --- | --- | --- | --- | --- | --- | --- | --- | --- | --- | --- |
| **Lake** | **^137^Cs (w)** | **^137^Cs (s)** | | **^90^Sr (w)** | | **^90^Sr (s)** | | **^241^Am (w)** | | **^241^Am (s)** | | **^239^Pu (w)** | **^239^Pu (s)** |
| Buryakovka | 0.1 | | 11 | | 0.45 | |  | |  | | 0.3 | 0 |  |
| Yampol | 0.23 | | 3.5 | | 0.22 | |  | |  | | 0.2 | 0 |  |
| Vediltsy | 0.15 | | 8 | | 0.3 | |  | |  | | 0.2 | 0 |  |
| Glinka | 0.22 | | 5 | | 0.45 | |  | |  | | 0.3 | 0 |  |
| Semikhody | 0.5-1.0 | | 90 | | 6.5-7.5 | |  | | 2.80E-03 | | 4 | 3.30E-03 |  |
| Krasnyansky | 0.5-1.5 | | 3700-7400 | | 14-28 | | 1480-3700 | |  | | 40-100 |  | 40-100 |
| Azbuchin | 3.3-3.6 | | 7500-20000 | | 80-500 | | 4000-7500 | | 8.00E-04 | | 100-200 | 2.00E-03 | 100-200 |
| Gluboke | 2.0-6.5 | | 550 | | 90-110 | | 200 | | 20-80E-3 | | 20 | 15-50E-3 |  |
